# Petersen’s Hernia in a Pregnant Woman Following Roux-en-Y Gastric Bypass Surgery: The Importance of Emergency Surgical Treatment

**DOI:** 10.7759/cureus.55815

**Published:** 2024-03-08

**Authors:** Citlali Calderón Espinosa de los Monteros, Agustin Castro Segovia, Steve Arciniega Belmont

**Affiliations:** 1 Department of General Surgery, Hospital Dr. Fernando Quiroz Gutierrez, Institute for Social Security and Service for State Workers (ISSSTE), Mexico City, MEX

**Keywords:** pregnant woman, mesenteric internal hernia, emergency exploratory laparotomy, petersen's hernia, roux-en-y gastric bypass (rygb)

## Abstract

Roux-en-Y gastric bypass (RYGB) patients are at risk of creating potential spaces for possible internal hernias during the procedure. During pregnancy, the pregnant uterus elevates the bowel, increasing intra-abdominal pressure. Cases reported to date have described mild abdominal pain and no evidence of peritoneal irritation, with inconclusive ultrasound and MRI findings for diagnosis of Petersen's hernia. We present the case of a 42-year-old female patient with a history of RYGB eight years earlier without complications, with a pregnancy of 34 weeks of gestation. Symptomatology began with colicky abdominal pain in the epigastric, with irradiation to the right upper quadrant. On physical examination, revealed a painful abdomen on the median and deep palpation in the epigastric and right upper quadrant, the rest of the studies were inconclusive. As there was no improvement of the symptoms in 12 hours, an emergency diagnostic laparoscopy was performed, finding a strangulated Petersen's hernia requiring resection, with the closure of the gastric pouch, intestinal anastomosis, and Stamm gastrostomy with closure of the mesenteric gap. Therefore, a pregnant patient presenting with upper quadrant abdominal pain with a history of RYGB, even one with normal labs and imaging, should be assumed to have an internal hernia until proven otherwise. The emergency surgical approach is associated with early resolution and prevents its progression with catastrophic results.

## Introduction

Maternal obesity is defined as a pre-pregnancy body mass index (BMI) ≥ 30 kg/m^2^, this condition increases the maternal risk of presenting with gestational diabetes, preeclampsia, postpartum hemorrhage, and termination of pregnancy by cesarean section. For the newborn, there is an increased risk of being small or large for gestational age, pre-term or post-term pregnancy, congenital anomalies such as neural tube defects, and perinatal mortality [1,2]. Weight loss following bariatric surgery reduces cardiovascular risks, improves fertility, and reduces obesity-related complications during pregnancy [3].

Bariatric surgery increases postoperative conception rates by 33% to 100% secondary to an improvement in hormonal regulation with restoration of the menstrual cycle and ovulation [2]. In addition, bariatric surgery is the most effective treatment for long-term weight loss; most surgeries are performed on women of reproductive age [1]. However, bariatric surgery also carries long-term complications, with an increased risk of severe maternal and fetal morbidity and mortality [3].

Roux-en-Y gastric bypass (RYGB) is a malabsorptive procedure because part of the small intestine is bypassed. This surgery is very effective for weight loss; however, it has the risk of re-operation in 3% to 20% of patients, secondary to complications such as anastomotic stenosis, intestinal obstruction secondary to adhesions, abdominal wall hernias, intussusceptions, and internal hernias [4].

Emphasizing the diagnostic challenge of finding an internal hernia after RYGB, the literature states that there will be a catastrophic outcome if the diagnosis is missed and timely treatment is not given, high diagnostic suspicion should be raised when a patient has a history of RYGB and severe epigastric pain or umbilical pain [4].

Of concern are the high rates of maternal (9%) and fetal (13.6%) deaths related to small bowel obstruction and necrosis secondary to internal hernia [4].

Therefore, we conducted a literature review and impact evidence recommendations on the safe management of a Petersen's hernia (PH) in a pregnant person to avoid its complications. We present the case of a pregnant patient with a history of RYGB, and a diagnosis of PH complicated with intestinal necrosis, emphasizing the importance of timely surgical treatment.

## Case presentation

The case presented is of a 42-year-old female patient who was previously admitted to the gynecology emergency department with a history of obesity grade 3 (WHO) treated with RYGB eight years earlier without complications, currently with a BMI within normal ranges, curettage four years earlier without complications, cervical cerclage four months earlier without complications, and with 34 weeks gestation and advanced maternal age.

The patient began with symptoms referring to colicky abdominal epigastric pain with irradiation to the right upper quadrant triggered by fatty foods, intensity 7/10 on the visual analog scale, accompanied by nausea and vomiting, for which she was admitted to the gynecological emergency department.

Her vital signs were within normal parameters. A physical examination was carried out, finding the abdomen at the expense of the pregnant uterus, with a single live product, in transverse position with a fetal heart rate of 153 beats per minute, no palpable uterine activity, a normal rate of peristalsis, soft, moderately painful to mid and deep palpation in the epigastric and right upper quadrant, negative Murphy's sign, negative appendicular points, negative rebound, and no evidence of peritoneal irritation. The rest of the examination was without alterations.

On admission, with laboratories within normal parameters. Liver and biliary tract ultrasounds were requested, finding the gallbladder with cholelithiasis, with no evidence of aggravation. Without having the magnetic resonance service, it was decided not to perform computed axial tomography due to the risk of radiation exposure.

The initial diagnosis included abdominal pain syndrome, which occurred at 34 weeks of pregnancy, and cholelithiasis was integrated.

When there was no improvement in symptoms within 12 hours, it was decided to admit her to the operating room to perform an emergency diagnostic laparoscopy.

The surgical findings were abundant free fluid and purulent fibrin clots in the abdominal wall and diaphragmatic side of the liver, in addition to the ischemic-necrotic distended small intestine.

During the diagnostic laparoscopy, the patient presented hemodynamic instability with hypotension of 80/60 mmHg, tachycardia of 110 beats per minute, and tachypnea > 22 breaths per minute. The pregnancy was terminated with a cesarean section. The newborn was alive, female, with a cephalic presentation, clear amniotic fluid, a weight of 1720 grams, a size of 41 centimeters, Apgar 6 of 8, and 35.2 weeks of gestation are calculated. The newborn was immediately taken to the intensive care unit.

**Figure 1:**
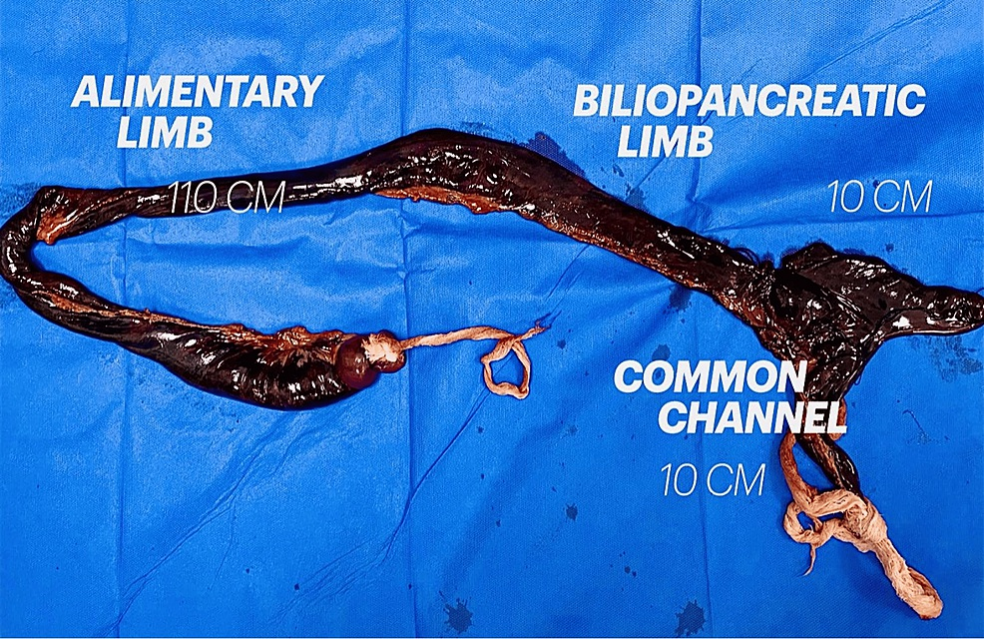
The figure shows intestinal necrosis of all Roux-en-Y loops secondary to strangulated Petersen's hernia, containing 110 cm of the alimentary limb, 10 cm of the biliopancreatic limb, and 10 cm of the common limb.

An exploratory laparotomy was performed. The surgical findings revealed free liquid in the peritoneal cavity, characterized by a murky and fetid appearance, with a volume quantified at 1500 cm^3^. Additionally, an intestinal loop was observed exhibiting distension and necrosis. Strangulated PH contained 10 cm of biliopancreatic limb, 110 cm of alimentary limb, and 10 cm of common limb (Figure 1).

There were 40 centimeters of biliopancreatic loop, gastric pouch, 30 centimeters of jejunum, and 150 centimeters of ileum, colon, and rectum, with no evidence of vascular compromise.

Intestinal resection was performed with gastric pouch closure, intestinal anastomosis, and a Stamm gastrostomy with mesenteric gap closure. Bleeding of 1050 cm^3^ was reported during the operation, so four globular packs and three fresh frozen plasmas were transfused.

Three days later, the patient presented fever (38.1°C), tachycardia with 110 beats per minute, and tachypnea of 24 breaths per minute, as well as leukocytosis of 13.87 x 10^3/μL. Washing of the abdominal cavity with abscess drainage was performed, and one week later placement of a decompressive gastrostomy tube in the gastric pouch was performed.

The patient was discharged from the hospital after one month of hospitalization because of clinical improvement.

The patient presented with chronic malnutrition, so it was decided to refer her to the bariatric service in another hospital unit. One year later, a RYGB restoration was performed, with gastro-gastric anastomosis of the posterior face of the gastric pouch with the anterior face of the stomach and a reconstitution of the transit with a gastro-jejunal anastomosis, with tolerance to diet and favorable evolution.

Follow-up is continued in our hospital unit, diagnosing a giant incisional hernia, which is kept under surveillance due to the patient's refusal to perform a surgical procedure.

## Discussion

In 1900, Petersen described the space created between the transverse mesocolon, the Roux branch, and the retroperitoneum dorsally. PH is created iatrogenically after each type of gastrojejunostomy [5].

In the meta-analysis by Apostolou et al. [5], they report an overall incidence of PH of up to 14.3% regardless of pregnancy, mentioning that there is a bias in its true incidence, since not all cases are reported, but mainly asymptomatic cases with incidental findings [5].

As the uterus increases in size, it pushes the bowel upward, increasing intra-abdominal pressure and increasing the risk of a PH, with a reported incidence of 8% to 10% in pregnant patients [2], being more frequent in the third trimester of pregnancy [6].

Pregnancy should be delayed for 12 to 24 months following bariatric surgery while weight stabilizes [2]. Pregnancy and rapid weight loss within the first year after bariatric surgery are associated with higher rates of preterm delivery, delivery of a small-for-gestational-age newborn, and admission to the neonatal intensive care unit compared to pregnancies more than two years after bariatric surgery [4]. This weight loss occurs with greater speed during the first year [5], so in the clinical case presented, the main risk factor was the increase in intra-abdominal pressure, since the RYGB was performed eight years before pregnancy.

The clinical picture of PH varies widely, from asymptomatic, mild, intermittent abdominal pain to presenting with evidence of peritoneal irritation secondary to acute small bowel obstruction, which may result in strangulation with intestinal necrosis, requiring an emergency exploratory laparotomy [5,2]. Dave et al. [7], in a systematic review of 59 pregnant patients with a history of RYGB and a diagnosis of internal hernia, found that the most common presenting symptom was epigastric pain with nausea and vomiting [7].

Fisher et al. [4] mention that "A pregnant patient presenting with acute abdominal pain and a previous RYGB even if she has normal laboratory and imaging results, has an internal hernia until proven otherwise" [4].

Pregnant patients with a history of RYGB and a diagnostic suspicion of internal hernia should be admitted to a hospital unit and evaluated by a bariatric surgeon. If the pain disappears during fasting but reappears after starting food, diagnostic laparoscopy should be considered, but if the pain continues despite fasting, emergency exploratory laparotomy should be performed to minimize the risk of progressive intestinal necrosis and associated maternal-fetal morbidity [4]. An open or laparoscopic approach can be safely performed during any trimester of pregnancy; Gonzalez et al. [6] recommend open surgery during the third trimester of pregnancy, due to a reduced abdominal cavity [6].

CT does not always confirm the diagnosis of PH in pregnant patients after a RYGB so at the slightest diagnostic suspicion, it is recommended to perform diagnostic laparoscopy [1].

However, CT with intravenous contrast is the imaging study of choice. A CT scan of the abdomen and pelvis rarely exceeds 25 mGy, and the risks to the fetus are negligible with doses lower than 50 mGy, so Gonzalez et al. [6] recommend performing the study, previously considering the risk-benefit for the couple on an individual basis. CT helps us to diagnose small bowel obstruction, intestinal ischemia, or perforation, which in turn helps us to improve surgical planning [6]. MRI eliminates the risk of radiation; however, it is not available in all hospitals, and the time factor is paramount.

Apostolou et al. [5] state that reported cases of PH after RYGB in a pregnant patient at present have been found with mild abdominal pain and no peritoneal irritation which, when complemented with ultrasound or magnetic resonance imaging (MRI), do not conclude the diagnosis.

Addressing the prevention of PH following RYGB closure of the mesenteric defect has been proposed; however, Apostolou et al. [5] confirm that there is still a lack of consensus in the literature on whether to perform closure. Arguments against are potential tension caused by the gastrojejunostomy, which may result in hematomas, injury to the mesenteric vessels, or even small bowel adhesions that may cause intestinal obstruction, as well as additional costs due to increased operative time and suture material. In addition, some studies have shown very low PH rates in patients with RYBG without Petersen's space closure [5,8].

However, these studies do not consider pregnant patients, who are at higher risk of PH, and as mentioned by Apostolou et al. [5], in these studies, an antero-colic and antero-gastric approach was performed, which further decreased the incidence of PH compared to the retro-colic approach.

Gonzalez et al. [6] mention that in the retrocolic technique, there are three sites where an internal hernia can occur: through the transverse mesocolon defect, through the mesenteric defect of the entero enterostomy and through the space between the mesentery of the Roux branch and the transverse mesocolon (Petersen's space). However, they also mention that in the ante colic approach, two defects are also created: the mesenteric entero enterostomy defect and Peterseń's space defect [6], therefore the risk of PH is still present.

Apostolou et al. [5] conclude that there are slightly lower rates of bowel obstruction in patients with ante colic gastrojejunostomy and close Petersen's defect. Closure of the mesenteric defect with nonabsorbable sutures also reduces the incidence of PH; the use of stapling for this closure is also associated with favorable long-term results [5]. Reinforcement of the sutured Petersen’s defect with the use of a Bio A (GORE® BIO-A®, Newark Delaware, USA) mesh has been also suggested as an effective method in terms of elimination of the risk of PH [5].

Quian et al. [8] also report a higher incidence of internal hernia in the group where mesenteric closure is not performed, recommending that the jejunal mesentery be closed during RYGB in patients, further concluding that closing the mesentery (especially the jejunal space) could be a therapeutic option to reduce the risk of internal hernia in elderly patients and the length of hospital stay after RYGB [8].

On the contrary, Gonzalez et al. [6] mention that "Closing mesenteric defects during the RYGB procedure may not be sufficient to prevent internal hernias, because when intra-abdominal fat decreases with weight loss, the spaces between the sutures may widen and the defects may partially reopen." Two of their patients had mesenteric defect closure during RYGB and still presented an internal hernia, which justifies their explanation [6].

Therefore, every pregnant patient after RYGB should be closely monitored before and during pregnancy by a multidisciplinary team that includes an obstetrician, bariatric surgeon, nutrition specialist, and family physician, thus optimizing nutrition and maternal health and reducing the risks of maternal and fetal complications [4,2,9-11].

## Conclusions

A pregnant patient presenting with upper quadrant abdominal pain with a history of RYGB, even with normal lab work and imaging studies, should be considered to have an internal hernia until proven otherwise. Whether the ideal treatment should be urgent laparoscopy after detection of the condition or brief surveillance that maintains a low threshold at the slightest suspicion of distress for urgent surgery is still debated. The laparoscopic or open surgical approach is associated with early resolution and avoids progression with catastrophic outcomes, requiring a multidisciplinary team before and during pregnancy, thus avoiding all possible maternal-fetal complications.
